# Advances in sepsis-associated liver dysfunction

**DOI:** 10.4103/2321-3868.132689

**Published:** 2014-07-28

**Authors:** Dawei Wang, Yimei Yin, Yongming Yao

**Affiliations:** 1 Department of Microbiology and Immunology, Burns Institute, First Hospital Affiliated to the Chinese PLA General Hospital, No.51 Fucheng Road, Haidian District, Beijing, 100048 China; 2 Department of ICU, Weihai Municipal Hospital, Weihai, Shandong, China

**Keywords:** Sepsis, liver, dysfunction, inflammation, jaundice

## Abstract

Recent studies have revealed liver dysfunction as an early event in sepsis. Sepsis-associated liver dysfunction is mainly resulted from systemic or microcirculatory disturbances, spillovers of bacteria and endotoxin (lipopolysaccharide, LPS), and subsequent activation of inflammatory cytokines as well as mediators. Three main cell types of the liver which contribute to the hepatic response in sepsis are Kupffer cells (KCs), hepatocytes and liver sinusoidal endothelial cells (LSECs). In addition, activated neutrophils, which are also recruited to the liver and produce potentially destructive enzymes and oxygen-free radicals, may further enhance acute liver injury. The clinical manifestations of sepsis-associated liver dysfunction can roughly be divided into two categories: Hypoxic hepatitis and jaundice. The latter is much more frequent in the context of sepsis. Hepatic failure is traditionally considered as a late manifestation of sepsis-induced multiple organ dysfunction syndrome. To date, no specific therapeutics for sepsis-associated liver dysfunction are available. Treatment measure is mainly focused on eradication of the underlying infection and management for severe sepsis. A better understanding of the pathophysiology of liver response in sepsis may lead to further increase in survival rates.

## Introduction

Sepsis reflects a systemic inflammatory syndrome in response to an infection and represents the leading cause of death in the intensive care unit.[1] The predominant cause of morbidity and mortality is the development of multiple organ dysfunction syndrome (MODS) with subsequent organ failure. During the process of sepsis, the liver plays an important role in defensive responses to scavenge bacteria and produce inflammatory mediators. But the liver also acts as a potential target of dysregulated inflammatory response. Sepsis-associated liver dysfunction is traditionally viewed as a late feature of critical illness, manifesting jaundice and hyperbilirubinemia. However, recent studies have revealed liver dysfunction as an early event in sepsis.[2] Liver dysfunction is not the most common form of organ injury encountered in septic patients; when it culminates into liver failure, it becomes a grave complication. Therefore, it is important to understand the pathophysiological changes that contribute to liver dysfunction associated with sepsis, which has been defined as the combination of cellular injury in addition to heightened inflammation. This review will focus on the pathophysiological alterations, clinical characteristics and therapeutic considerations of sepsis-associated liver dysfunction.

**Table 1:** 

Access this article online	
**Quick Response Code**:	**Website**: www.burnstrauma.com
	**DOI**: 10.4103/2321-3868.132689

## Key points

- Sepsis-associated liver dysfunction is usually attributed to systemic or microcirculatory disturbance.
- Four main cell types which contribute to the hepatic response in sepsis are Kupffer cells (KCs), neutrophils, hepatocytes and liver sinusoidal endothelial cells (LSECs).
- Kupffer cells release cytokines, reactive oxygen species, and nitric oxide (NO) which induce LSEC and hepatocyte injury.
- Neutrophils, which are recruited to the liver and produce pro-inflammatory cytokines and chemokines, may further injure LSECs and hepatocytes.
- Abnormalities in quantity, morphology, and function of LSECs developed during sepsis.
- Sepsis induces a profound alteration in the hepatic ability to transport bile acids and bilirubin into the hepatic canaliculi, thereby causing cholestasis.
- Sepsis-associated liver dysfunction can roughly be divided into hypoxic hepatitis and jaundice. The latter is much more frequent in the context of sepsis.
- Treatment measure is mainly focused on eradication of the underlying infection and management for severe sepsis.

## Pathophysiological changes in sepsis-associated liver dysfunction

### Systemic and microcirculatory disturbances

Sepsis-associated liver dysfunction is usually attributed to systemic or microcirculatory disturbance. Numerous studies have been shown that there is a consistent correlation of cardiac output and macrovascular hepatosplanchnic inflow in sepsis. In septic shock, splanchnic blood flow and cardiac output are increased but not sufficient to counterbalance the high demands for oxygen and the inability of liver cells to extract oxygen.[3] Vascular mechanisms of defense against portal blood flow reduction are also altered, especially the defective hepatic arterial response.[4]

Microvascular tissue perfusion in severe sepsis is often uncoupled from the systemic circulation. Redistribution of intrahepatic blood flow in concert with a complex interplay between sinusoidal endothelial cells, liver macrophages, and passing leukocytes lead to a decreased perfusion and blood flow velocity in the liver sinusoids.[5] Activation and dysfunction of the endothelial cell barrier elicits a significant recruitment of both leukocytes and platelets in the liver microvasculature. Subsequently, formation of microthrombi further enhances liver tissue ischemia and damage. Emerging evidence implies that lipopolysaccharide (LPS)-induced intrahepatic endothelial dysfunction and microvascular disorder could be prevented by simvastatin.[6]

Substances that regulate vascular tone, such as NO, hydrogen sulfide (H_2_S), endothelin-1 and carbon monoxide (CO), are highly active during sepsis. For example, inducible NO synthase (iNOS)-derived NO is considered to be a contributor to the blood-cell recruitment, and the strong upregulation of iNOS might contribute to a microvascular dysfunction and hepatic injury.[7] In contrast, endothelial NO synthase (eNOS) appears to play a protective role in liver microcirculation during polymicrobial sepsis. Sepsis-related increase in bilirubin level, international normalized ratio, and lipid peroxidation in liver tissue were significantly attenuated by the early neuronal NO synthase (nNOS) and delayed iNOS blockade.[8] Additionally, H_2_S contributes to microcirculatory dysfunction in the systemic as well as hepatic microcirculations due to a differential vasoactive function on presinusoidal and sinusoidal sites within the liver.[9]

### KCs

KCs are presumed to be the primary defense against portal bacteremia and endotoxemia. They prevent bacteria and endotoxins from entering the systemic circulation by removing them from the portal vein blood. However, in the case of sepsis, the inflammation cascade is strongly activated. Inflammatory response will not only kill the bacteria but can also damage the liver. Following excessive LPS stimulation, KCs release cytokines [tumor necrosis factor (TNF)-α, interleukin (IL)-1β, IL-6, IL-12 and IL-18], reactive oxygen species (ROS) and NO, which induce endothelial cell and hepatocyte injury.[10] Lipoprotein and teichoic acid derived from Gram-positive bacteria could also activate KCs and induce liver injury. Accordingly, KC depletion reduces hepatic inflammation and apoptosis in abdominal sepsis.[11] However, the lack of IL-10 in KC-depleted mice resulted in a detrimental systemic pro-inflammation and a significant reduction in survival.

TNF-α is believed to be crucially involved in the development of liver injury in septic complications. TNF-α can directly stimulate hepatocytes to induce IL-6 production, increases the activity of cysteinyl aspartate-specific protease (caspase)-3, and postulates as an important factor in the development of hepatocellular apoptosis.[12] TNF-α also activates neutrophils and increases gene expression for adhesive molecules, which, in turn, enhances neutrophil adherence to sinusoidal endothelial cells in the liver, thus contributing to neutrophil-mediated liver injury. It has been demonstrated that TNF-α and IL-1β can activate mitogen-activated protein kinase (MAPK) and decrease multidrug resistance protein (MRP)2 expression.[13] Since bilirubin, glutathione (GSH), and divalent bile acids are the main substrates for MRP2, it is likely that the functional down-regulation of MRP2 accounts for the impaired transport of these compounds into bile, resulting in a parallel backflow of bilirubin from the hepatocyte into the bloodstream culminating in hyperbilirubinaemia. There is a rapid upregulation of liver IL-6 messenger RNA after LPS exposure. IL-6 is produced by KCs, hepatocytes, and LSECs after LPS stimulation. In combination with IL-1β and TNF-α, IL-6 markedly enhances gene expression of acute-phase proteins (APP) via transcriptional activation.[14] IL-18, secreted by KCs, could activate both TNF-α and Fas ligand-mediated hepatocytotoxic pathways in LPS-induced liver injury.[15] Lastly, increased levels of ROS have been demonstrated in different models of sepsis and this disturbance may lead to DNA, protein, or lipid damage resulting in apoptosis and necrosis followed by cell death.

### Neutrophils

Accumulation of neutrophils is an early phenomenon in the liver during the development of sepsis. Neutrophil recruitment to the liver is the result of a complex interaction between neutrophils, sinusoidal endothelium, hepatocytes, and KCs. During the process of septic response, activated KCs release leukotriene B4 and TNF-α, which in turn attract blood neutrophils into the liver and activate them.[16] Apoptotic hepatocytes have also been shown to function as chemotactic signals, triggering neutrophil transmigration.[17] Upregulation of endothelial integrins and intercellular adhesion molecule (ICAM)-1 also promotes neutrophil migration in LPS-induced liver injury.[18] In contrast, heme oxygenase (HO)-1 in neutrophil attenuates its infiltration in liver during sepsis via the inactivation of p38 MAPK.

Once neutrophils enter the liver, they start several antibacterial activities by secreting proteolytic enzymes and ROS. Meanwhile, they can induce the production of pro-inflammatory cytokines and chemokines. These mechanisms play major roles in protecting against infection, but they may also injure endothelial cells and hepatocytes.[19] For example, a homeostatic balance exists between the formation of ROS and their removal by endogenous antioxidant scavenging compounds under physiological conditions. Continued or excessive production of ROS can be deleterious to mitochondria and other organelles.[20] O_2_-and non-oxygen-derived radicals, such as NO generated by activated neutrophils, could rapidly react with each other to form peroxynitrite (ONOO^−^), which is one of the highly reactive oxidant species. ONOO^−^ can deplete intracellular GSH levels, inhibit mitochondrial superoxide dismutase, and oxidize mitochondrial proteins, thereby leading to cascades that cause cellular apoptosis or necrosis in septic liver.

### Hepatocytes

Hepatocytes, via receptors for inflammatory mediators, regulate their metabolic pathway toward glycogenolysis, gluconeogenesis, amino acid uptake, ureagenesis, as well as increased synthesis of coagulant factors, complement factors, and acute-phase proteins, all of them prioritizing protein synthesis and tissue repair. Simultaneously, the hepatic production of albumin, properdin, high-density lipoprotein, protein C and antithrombin decreased. During the metabolic changes in hepatocytes, KCs are believed to be a key regulator through synergistic effects of TNF-α, IL-1, and IL-6.[21] In addition, exogenous mediators, notably LPS, have direct and indirect cytotoxic effects on hepatocytes and trigger metabolic changes in hepatocytes.

APP (e.g., C-reactive protein, α1-antitrypsin, α2-macroglobulin, and thrombin-activatable fibrinolytic inhibitor) contributes to the pro-coagulant state. For instance, elevated C-reactive protein levels promote the expression of tissue factor by mononuclear cells, increase liver production of thrombin-activatable fibrinolytic inhibitor, and enhance fibrinolysis inhibition.[22] Neutrophils are also a source of tissue factor, and they are implicated in direct activation of the coagulation cascade in the early phases of sepsis. Study shows that Ca^2+^ channel blockers curb the sepsis-induced acute phase response by preventing sepsis-related hepatic Ca^2+^ changes and thus modulate the metabolic response of hepatocytes.[23]

Sepsis induces a profound alteration in the hepatic ability to transport bile acids and bilirubin into the hepatic canaliculi, thereby causing cholestasis. Visualization of the hepatobiliary excretory function in septic animals demonstrated a profound impairment of hepatocellular transport, specifically at the canalicular pole.[24] LPS induces KCs to release pro-inflammatory cytokines which lead to downregulation of transporters involved in bile flow, coordinated by nuclear receptors and transcription factors.[25] The transporters that are downregulated include MRP2, organic aniontransporting polypeptides (OATPs), bile salt export pump (BSEP), multiple drug resistance (MDR)-1 transporter, Na^+^, K^+^-ATPase and sodium-dependent taurocholate cotransporting polypeptide (NTCP). Reduced basolateral bile acid uptake appears to be related to a reduction in the expression of NTCP and OATPs. Impaired excretion of biotransformed molecules from the hepatocyte into the bile canaliculi results from a downregulation of canalicular bile acid transporters, including BSEP and MRP2.

In addition, the function of bile acid transporters can be impaired by reduced sinusoidal perfusion and depletion of ATP content, pointing to alterations in energy availability as a mechanism of the biotransformation dysfunction during sepsis. The defective expression of hepatocyte aquaporin-8, a water channel involved in bile secretion, also contributes to the development of bile secretory dysfunction in sepsis.[26] P-selectin-mediated recruitment of leukocytes plays a potential role in reduction of bile flow in sepsis-associated cholestasis.[27] Moreover, epithelial tight junctions in the hepatobiliary system are important in maintaining the osmotic gradient for bile production. In the development of severe sepsis, NO secretion could increase tight junction permeability in hepatocytes by decreasing zona occludens, which were key proteins to maintain tight junctions. Taken together, excessive formation of NO has been implicated in playing a role in cholestasis during sepsis.[28]

Apart from impaired hepatobiliary transport, a defect in phase I detoxification machinery, including cytochrome P450 (CYP450) enzymes (i.e., P450 family CYP1, CYP2 and CYP3) and phase II conjugating enzymes (glutathione-S-transferases, bilirubin-UDP-glucuronyl transferase), would result in non-detoxification of substances that are normally bile-excreted. The downregulation of CYP enzymes is due to the reduction of aryl hydrocarbon receptor (AhR) and AhR nuclear translocator (Arnt), two critical transcription factors involved in the regulation of CYP1A2 mRNA.[29] Furthermore, AhR and Arnt expressions are inversely correlated with pro-inflammatory cytokines in sepsis. In addition to pro-inflammatory cytokines, NO may contribute to the suppression of CYP in sepsis via the interplay of two different mechanisms: NO-dependent suppression of protein via the enhanced iNOS, and NO-dependent transcriptional suppression via eNOS.[28] This suppression could be reversed by administration of an NO inhibitor.

As for the cellular signal transduction of impaired biotransformation, phosphatidylinositol 3-kinase (PI3K) is a pivotal molecular switch.[30] PI3Kγ^−/−^ mice show preserved morphology and function of the bile canaliculi in sepsis. PI3K signaling also affects hepatic phase I and II metabolism of bile acids. Interestingly, neutrophil recruitment into septic liver is completely prevented in PI3Kγ^−/−^ mice.

Autophagy is a protective molecular pathway in the setting of sepsis. HO-1-mediated autophagy occurs transiently in hepatocytes at an early stage during sepsis.[31] The initial elevation of autophagy may reflect the early host response to oxidative stress as well as mitochondrial damage caused by septic insult, and protect against hepatocellular death in early stages of sepsis. However, the decline in autophagy at late sepsis may cause insufficient recycle function, which contributes to hepatic failure. Additionally, altered membrane fluidity and G1 cell cycle arrest in hepatocytes have been reported in animal models of severe sepsis.

### Liver sinusoidal endothelial cells

Abnormalities in quantity, morphology, and function of LSECs have been reported during experimental sepsis. In response to sepsis, LSECs undergo Fas-induced apoptosis, culminating in a decrease in overall cell number and an increase in liver tissue permeability. KCs could protect LSECs from further injury by downregulating gp130 expression on LSECs.[32] In contrast, KCs could potentiate LSEC injury by ligating programmed cell death ligand-1.[33] LSECs have the capacity to produce immunoregulatory and pro-inflammatory cytokines (e.g., NO, IL-1 and IL-6). This production is increased when LSECs are treated with LPS.[34] LSECs also function as antigen-presenting cells for CD4^+^ T cells, and LPS downregulates CD4^+^ T cell activation by antigen-presenting LSECs. In addition, LSECs are perforated with fenestrations, pores that facilitate the transfer of lipoproteins and macromolecules between blood and hepatocytes. In Gram-negative bacterial sepsis, defenestration of LSECs by bacterial toxins was involved in the pathogenesis of hyperlipidemia.[35] Intravenously injected LPS in rats resulted in LSECs enlargement, sieve plate disruption, and gap formation.

### Other hepatic responses to sepsis

Chronic sequelae of experimental sepsis are characterized by abscess formation, persistent inflammation, and substantial liver injury as well as fibrosis. The latter is associated with increased number of hepatic stellate cells and deposition of collagen types I and III.[36] Moreover, significant temporal loss of hepatic B cells was seen following LPS challenge. Histological examination shows that hepatitis and steatosis are the main findings in the liver in the majority of patients dying from sepsis.

### Sepsis-associated liver dysfunction and MODS

When sepsis-associated liver dysfunction progresses to acute liver failure, a cascade of serious complications (e.g., cerebral edema, coagulopathy, cardiovascular instability, respiratory failure and renal failure) can occur. In another word, sepsis-associated liver dysfunction could induce the development of MODS.

Firstly, superfluous ammonia in acute liver failure (ALF) patients is converted to osmotically active glutamine, producing osmotic cerebral edema. Impaired cerebral blood flow autoregulation, systemic inflammatory response, and ischemic injury have also been proposed as the cause of cerebral edema.[37] Secondly, deficiencies of fibrinolytic proteins, anticoagulant proteins and pro-coagulation factors are often present in liver failure; in part due to failure of synthesis as well as consumption of these factors. Quantitative and qualitative platelet dysfunction is also shown in ALF. Thirdly, increased NO production and cyclic guanosine monophosphate (GMP) may be involved in cardiovascular instability of ALF patients. Fourthly, direct drug nephrotoxicity, acute tubular necrosis, and the development of abdominal compartment syndrome are common causes of renal impairment in ALF.[38] Lastly, acute lung injury/acute respiratory distress syndrome (ARDS) is not uncommon in ALF; particularly, there is a requirement for vasopressors and concurrent intracranial hypertension.

## Clinical characteristics of sepsis-associated liver dysfunction

Some data showed that liver dysfunction occurs in 34.7% of septic patients.[39] Nevertheless, the incidence of liver dysfunction remains varied because current diagnostic tools are lacking, notably those can detect the early liver insults. In septic patients, liver dysfunction can vary from subclinical injury to overt failure.[40] Sepsis-associated liver dysfunction can roughly be divided into hypoxic hepatitis and jaundice. Hepatic failure is traditionally considered a late manifestation of sepsis-induced multiple organ failure. It is rare if the underlying process is detected and adequately treated.

### Hypoxic hepatitis

Hypoxic hepatitis is related to hypoperfusion in the presence of hypovolemia as well as inadequate cardiac output, and it resolves fast under supportive therapy.[41] The leak of transaminase enzymes is characteristic of this injury and reflects acute cellular and mitochondrial injury. Aspartate aminotransferase (AST) and alanine aminotransferase (ALT) are rapidly and markedly elevated after an episode of hypotension or shock, with the predominance of AST over ALT, along with minimal elevation of gamma-glutamyltransferase (GGT). AST as well as ALT levels were decreased rapidly in a few days once the underlying course was corrected. Septic shock with hypoxic hepatitis can result in fulminant hepatic failure, which frequently leads to disseminated intravascular coagulation and bleeding. Lactate and amino acid clearance, as well as protein synthesis are reduced. Gluconeogenesis and glycogenolysis are decreased, and hypoglycemia can occur.[42]

### Jaundice

The incidence of jaundice was reported to be 34% in septic patients and the overall mortality rate of these patients was 61%. Jaundice is much more frequent than hypoxic hepatitis in sepsis. Commonly, jaundice is a late event in the course of severe sepsis. However, jaundice could be experienced at an early stage of sepsis even in the absence of fever or leukocytosis.[43] Both Gram-negative and Grampositive bacterial infections could lead to jaundice. In most instances of sepsis-induced jaundice, the infection is intraabdominal and may include biliary infection, urinary tract infections, or intra-abdominal abscesses. Notably, jaundice has also been reported to be associated with pneumonia, meningitis, and bacterial endocarditis. Cholestatic liver dysfunction is characterized by (supra-) normal tissue perfusion. In addition, the elevation of conjugated bilirubin with disproportionately low elevations of AST, ALT, and biliary enzymes is characteristic.[44] Liver histological studies showed a predominant intrahepatic cholestasis, which was reversible once the infection was controlled.

## Prognosis of sepsis-associated liver dysfunction

Sepsis-associated liver dysfunction often resolves in parallel with the remission of sepsis. In contrast, the lack of baseline liver dysfunction resolution or the development of new liver dysfunction during the first week of sepsis or both were associated with a lower 28 day survival rate.[45] The persistence or development of liver failure in the 72 h period after the onset of severe sepsis was strongly associated with a poor outcome.

Serum bilirubin is the most widely used biomarker to diagnose hepatic dysfunction during sepsis. In a large Austrian multicentric cohort, early increase in plasma bilirubin (>2 mg/dl), noted in approximately 10% of critically ill intensive care patients and many of whom were septic, was a strong independent risk factor for subsequent mortality. After multivariable adjustment for potential confounding factors, elevated serum bilirubin levels within 72 h of admission were associated with an increased risk of mortality in patients with severe sepsis and septic shock.[46] However, bilirubin level lacks specificity to reflect the full spectrum of liver dysfunction and differentiate an acute response from a preexisting organ chronic disease.

Recently, it was reported that levels of both conjugated and unconjugated chenodeoxycholic and taurodeox ycholic acid were increased in septic patients on the day of diagnosis, and these increased levels showed a stronger correlation with 28 day mortality than did bilirubin levels.[30] Similarly, plasma disappearance rate of indocyanine green (PDR_ICG_) could detect septic liver dysfunction earlier than changing in bilirubin.[47] This indicator seems to correlate with outcome of patients but lacks information about liver-bile interactions, thereby it fails to evaluate specifically canalicular transport, which is an increasingly recognized component of sepsis-associated excretory dysfunction.

Patients aged 61 years or older were prone to sepsis-associated liver injury, whereas it was rare in patients aged 15–60 years.[39] Age-related host immunological potency, malignant neoplasms, diabetes mellitus, and cerebrovascular diseases might be potent risk factors in the elderly. Interestingly, development of jaundice in elderly patients with bacterial sepsis was associated with increased survival. This result may represent robust immunity in elderly patients with sepsis, which might be responsible for their survival.

In the pathophysiological alterations of sepsis, hepatic inflammatory injury depends on the hormonal milieu. A higher susceptibility of liver injury in male individuals and protection against liver injury with female hormones has been reported in septic animals. As for septic patients, acute liver injury was significantly higher in males than in females, and the mortality in males was higher than in females.[48] CYP activity, reflected by the aminopyrine breath test, is also a clinically useful tool for predicting outcome in the early stages of sepsis.[49] CYP activity returned to normal levels in the survivor patients while they remained low in the non-survivors at the late phase of sepsis.

## Therapeutic considerations for sepsis-associated liver dysfunction

No specific therapeutics for sepsis-associated liver dysfunction are currently available. Treatment measure is mainly focused on eradication of the underlying infection and management for severe sepsis.[50] Early antibiotic therapy and infection source control are essential in improving liver function. In addition, an appropriate hemodynamic support permits the restoration of liver perfusion, and it is a critical step in preventing liver damage. Thus, hemodynamic support as recommended is critical (i.e., fluid resuscitation, vasopressors, inotropic therapy, and corticosteroids). During the process of sepsis-related organ dysfunction, good management of extrahepatic organ dysfunction is also beneficial in improving liver function.

GSH is widely used as a hepatoprotective drug during sepsis in some area. During the initial phase of sepsis, as represented by an intravenous LPS challenge to healthy volunteers, plasma concentrations of total GSH decreased. Accordingly, GSH or trophic feeding of GSH precursors amino acid during parenteral nutrition protects against peroxidative damage in septic animals.[51] However, clinical studies are needed to confirm the benefit of GSH, and we do not recommend using the drug in the treatment of sepsis-associated liver dysfunction.

It is noteworthy that altered biotransformation of endobiotics and xenobiotics is an important aspect of perpetuated liver damage in sepsis.[24] In addition, considering hypoglycemia is a frequent event in liver failure, the close monitoring of blood glucose levels and careful glucose administration are required, and intensive insulin therapy should then be used with caution.

Some experimental and clinical studies provide important insights into specific treatment of sepsis-associated liver dysfunction. Hepcidin protected against LPS-induced liver injury in septic animals,[52] and it significantly decreased hepatic pro-inflammatory cytokine expression and liver injury, leading to reduction of early lethality. Enhanced autophagy and reduced apoptosis might also account for the protective effects of hepcidin. CO_2_ pneumoperitoneum could suppress hepatic TNF-α and IL-6 expression in sepsis.[53] Histological analysis showed a reduced inflammatory infiltration in liver in animals subjected to CO_2_ pneumoperitoneum. Adiponectin (APN), which is an adipose tissue-derived hormone, is known as an anti-inflammatory cytokine. APN deficiency elicits the production of inflammatory mediators, including TNF-α, IL-6 and monocyte chemoattractant protein (MCP)-1 and aggravates sepsis-induced hepatic injury. Administration of rosiglitazone, which increased the plasma APN concentration, significantly lowered plasma levels of inflammatory mediators, including TNF-α, IL-6, and MCP-1 during sepsis.[54]

Hyperoncotic albumin has a beneficial effect on liver injury and survival in rats with peritonitis-induced sepsis.[55] The increased plasma IL-1β, IL-6, nitrite/nitrate concentrations, liver iNOS expression, and liver superoxide levels in septic rats were attenuated after treatment with hyperoncotic albumin. Calcium, a major second messenger in several cellular signaling events, is required by the KCs for the generation of iNOS. Under endotoxemic conditions, calcium channel antagonists inhibit LPS-mediated iNOS expression by KCs, accompanied by a limitation of hepatocellular injury.[56]

Montelukast, a leukotriene receptor antagonist, abrogates LPS-induced response of liver injury and suppresses the release of inflammatory as well as oxidative stress reaction via its antioxidant properties and enhancement of enzymatic antioxidant activities.[57] Flunixin-meglumin, a well known non-steroidal anti-inflammatory drug (NSAID), has antioxidant properties and can be used to attenuate liver damage in LPS-induced sepsis.[58] Superoxide dismutase, which dismutates superoxide to hydrogen peroxide, has also been shown to offer protection against LPS-induced liver injury.

Some evidence supports the concept that improving the microcirculation may prevent or ameliorate sepsis-induced liver dysfunction. Pretreatment with dobutamine improves survival rate, liver function and hepatic microcirculation in septic rats via β1-adrenoceptor activation, in addition to its circulatory mechanism.[59] Dexmedetomidine, a novel lipophilic imidazole derivative with a high affinity for α2-adrenoceptors, has a protective effect on liver injury during experimental sepsis. It attenuates central venous congestion, hepatic sinusoids congestion and dilation, and portal tract inflammation.[60]

Both liver perfusion and hepatic inflammatory response in sepsis might be affected by sympathetic nerve activity. Thoracic epidural anesthesia, which is associated with regional sympathetic block, reverses sepsis-induced hepatic hyperperfusion and reduces leukocyte adhesion in the liver of septic rats.[61] Similarly, modulation of the sympathetic nervous system by blocking α2A-adrenoceptor significantly reduced serum levels of pro-inflammatory cytokines, chemokines, liver enzymes (AST and ALT) and lactate.[62] In contrast, some catecholamines, mimicking sympathetic nerve activity, can induce an inflammatory response and exacerbate the hepatic dysfunction observed during sepsis.

Several studies showed the benefit of antithrombin III supplementation therapy for patients with low antithrombin III activity in severe sepsis. But the response of antithrombin III activity after supplementation decreases in proportion to the severity of sepsis and liver dysfunction.[63] Liver X receptor (LXR) is a transcription factor of the nuclear receptor family, regulating genes involved in metabolism, inflammation, and apoptosis. LXRa plays a protective role in the development of liver injury in experimental sepsis.[64] Therapeutic hypothermia attenuates liver injury in polymicrobial sepsis by enhancing the Akt signaling pathway and inhibiting apoptosis.[65]

Finally, extracorporeal supportive devices have been advocated to replace the liver function in liver failure; however, the complexity of liver metabolic, synthetic, detoxifying and excretory functions makes the extracorporeal hepatic support extremely difficult. So far, no survival benefit could be demonstrated compared with standard medical therapy.[66]

## Conclusion

Hepatic dysfunction may be a result of repeated infection or shock, overactivation of the systemic response, persistent microcirculatory failure, or even undesirable side effects of the treatment provided. Early identification of sepsis-associated liver dysfunction and early management of severe sepsis as recommended are crucial to improve the outcome. The precise cellular basis of sepsis-associated liver dysfunction is not completely understood and its consequences remain largely unknown. A better understanding of the pathophysiology of hepatic response to sepsis will lead to further improvement in survival rates.

## Announcement

### 17^th^ Congress of the International Society for Burn Injuries (ISBI)

Sydney, Australia, 2014, 12th–16th October Website: http://isbi2014.com
